# Imaging review of aortic fistulas

**DOI:** 10.1007/s00261-026-05544-8

**Published:** 2026-05-09

**Authors:** Divij Agarwal, Christine O. Menias, Mahan Mathur, Sumit Dhital, Archana Laroia, Hardik U. Shah, Yashant Aswani

**Affiliations:** 1 Institute of Liver and Biliary Sciences, New Delhi, India; 2 Mayo Clinic Hospital, Phoenix, USA; 3 Yale University, New Haven, USA; 4 University of Iowa Health Care, Iowa City, USA; 5 Emory University, Atlanta, USA

**Keywords:** Atherosclerosis, Aortic aneurysm, Endovascular aortic repair, Aortoesophageal fistula, Aortoenteric fistula, Aortocaval fistula, Aortopulmonary fistula, Aortobronchial fistula, Aortoatrial fistula

## Abstract

Aortic fistulas represent rare but life-threatening communications between the aorta and the adjacent structures, most commonly the gastrointestinal tract. They are classified as primary, arising spontaneously in the setting of a native, diseased aorta, or secondary to prior aortic surgery or endovascular repair. Clinical presentation is often variable and nonspecific—ranging from gastrointestinal bleeding, sepsis, abdominal pain to hemodynamic collapse—making imaging pivotal for diagnosis. Computed tomography angiography is the preferred imaging modality. Imaging features such as visualization of the fistula tract and active contrast extravasation into the fistulized hollow organ are definitive signs of aortic fistulas, but are rarely encountered. On the contrary, nonspecific indirect imaging features such as loss of fat planes and/or ectopic foci of gas are more frequently seen. Prompt recognition of these indirect imaging features is crucial, as delayed diagnosis significantly increases mortality. This review article summarizes the types, clinical features, and imaging findings of aortic fistulas, emphasizing the radiologist’s pivotal role in timely detection and management of aortic fistulas.

## Introduction

Aortic fistulas are abnormal vascular communications between the aortic lumen (high intraluminal pressure) and nearby structures. They are extremely uncommon and require prompt diagnosis and urgent intervention, due to the risk of rapidly fatal hemorrhage [1]. Aortic fistulas (AoFs) most commonly arise secondary to previous surgical or vascular interventional procedures or trauma [2]; however, they can also develop spontaneously in diseased aorta, such as aorta affected by advanced atherosclerosis or blood-borne systemic infection. Other infrequent causes of AoFs include malignancy, radiation-induced vascular injury, vasculitis, and collagen-vascular diseases. Establishing the diagnosis of AoFs in the absence of any history or intervention can be difficult and may be overlooked. In recent years, the incidence of AoFs has risen, reflecting both the growing prevalence of atherosclerotic disease and more frequent use of endovascular graft aortic repair [3]. While AoFs may be asymptomatic initially, affected patients can present with hematemesis, melena, hemoptysis, hematuria, or abdominal/chest pain, depending on the organ or structure communicating with the aortic fistula.

Owing to the rarity of AoFs, nonspecific clinical presentation, and the potential hazards of invasive diagnostic methods, imaging serves as the cornerstone for identifying and establishing the diagnosis, with CT angiography (CTA) being the preferred imaging modality. Direct imaging features can help establish the diagnosis with high certainty (Table 1) [4]. These include visualization of the fistula tract, contrast extravasation from the aorta into the fistulized structure, leakage of orally administered gastrointestinal (GI) contrast into the periaortic space, and graft migration into a communicating organ. However, these direct features may not always be present. More commonly, indirect or non-specific imaging features **(**Table 1**)** may be present, such as increased perigraft soft tissue and/or fat stranding, bowel wall thickening, aortic mural hematoma, ectopic air in the aorta or suspected tract, or irregularities of the interface between the aorta and the fistulizing structure [4, 5].

**Table 1:** Imaging features of aortic fistulas

	Direct imaging features	Indirect imaging features
Frequency	Less common	More common
Imaging features	1) Direct visualization of the fistulous tract 2) Extravasation of intravenous contrast from the aorta into the fistulizing organ 3) Oral contrast leaking into the periaortic and/or paraprosthetic space 4) Aortic graft migration into the adjoining fistulized structure	1) Abnormalities of the aortic wall: ¬ Aortitis ¬ Aneurysm or pseudoaneurysm formation ¬ Aortic mural thrombus ¬ Malpositioned graft ¬ Discontinuous plaque 2) Irregularity of the interface between the aorta and the adjoining fistulizing organ: ¬ Loss of intervening fat planes ¬ Periaortic air foci ¬ Abnormally thickened or tethered walls of the fistulizing organ 3) Others: ¬ Air within the aortic lumen ¬ Peri-aortic collection/hematoma ¬ Unexplained free hemorrhage

## Classification

AoFs can be classified as primary and secondary (Table 2): Primary AoFs occur in a vessel with no prior surgery/intervention, and are most commonly due to atherosclerosis [3]. Secondary AoFs are more common and develop after aortic surgery/intervention. For instance, secondary aorto-enteric fistulas are commonly encountered due to an increase in abdominal aortic aneurysm (AAA) repair surgeries and stent graft placements [2].

**Fig. 1:**
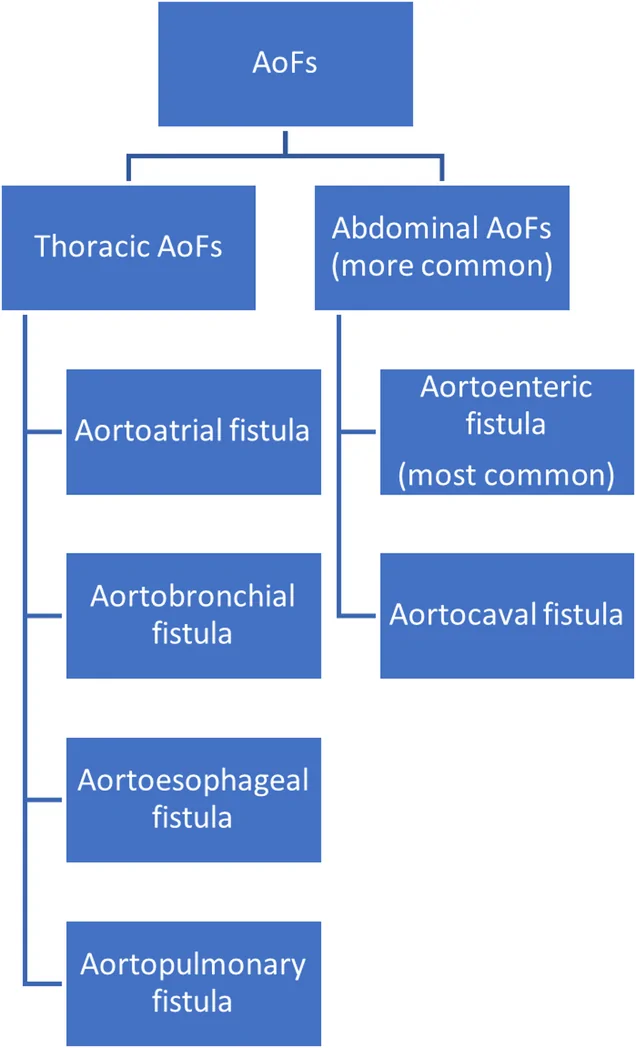
Classification of aortic fistulas (AoFs) according to the part of the aorta involved

AoFs can also be classified based on the part of the aorta involved, i.e., thoracic or abdominal aorta (Fig. 1).

**Table 2:** Differences between primary and secondary aortic fistulas [3, 6, 7]

	Primary aortic fistula	Secondary aortic fistula
Definition	Spontaneous fistulous communication between the native aorta and the adjacent structure without prior aortic surgery	Fistulous communication occurring after a prior aortic surgery or endovascular repair
Relative frequency	Rare	More common than the primary type
Clinical context	1) Aortic disease • Atherosclerotic aneurysm • Aortic dissection • Penetrating atherosclerotic ulcer	1) Graft infection 2) Graft migration, kinking, or structural failure 3) Chronic perigraft inflammation 4) Pressure necrosis from the graft 5) Endotension with the abdominal aortic aneurysmal sac 6) Coil embolization of endoleak post endovascular abdominal aortic aneurysmal repair
Risk factors/catalysts (common to both types)	1) Infection • Mycotic aneurysm/infected aneurysm • Tuberculosis • Syphilis (rare) 2) Inflammatory • Vasculitis • Radiation • Adjacent malignancy 3) Trauma • Blunt or penetrating trauma • Instrumentation (rare)	

### Imaging modalities

#### CT angiography

CT Angiography (CTA) is considered the initial imaging modality of choice in view of its widespread availability, short acquisition time, and high spatiotemporal resolution [2]. It can detect bleeding rates as slow as 0.3–0.5 mL/min. Three-dimensional volume rendering technique (VRT) images provide detailed anatomical information and help plan surgery or interventions. A specific CT protocol may be difficult to implement for AoFs due to non-specific clinical presentation. However, a multiphasic acquisition is the key. A non-contrast phase helps assess hemorrhage, intramural hematoma, surgical material, and vascular calcifications. A virtual non-contrast (VNC) image reconstructed from the arterial phase of a dual-energy CT is a viable alternative. Although the VNC image is usually noisier than a true non-contrast image, it is usually of sufficient diagnostic quality [8]. An arterial phase and an optional delayed phase (120 s) should be obtained by using automated bolus tracking, with the region of interest placed in the descending aorta. The delayed phase helps detect flow in the fistulous tract or accumulation of contrast within connecting structures [9], and should be acquired when there is a clinical suspicion of AoFs, or when imaging a patient with an aortic graft [10].

When assessing the aortic root or ascending aorta, the protocol should be optimized with cardiac gating techniques [3]. This becomes crucial to detect fistulous communications between the aorta and the atrium and between the aorta and the pulmonary artery, which may be momentarily identified as jets of contrast blush. If the heart rate is greater than 75 beats/min, a beta-blocker may be used, unless contraindicated.

Positive oral contrast should be avoided as it can obscure the extravasation of vascular contrast into the bowel, which is an important sign of AoFs [4]. However, in clinically stable patients, neutral oral contrast may be administered, along with intravenous contrast, for better detection of vascular-to-bowel contrast medium extravasation [3].

#### MR angiography

MR Angiography is useful in patients with renal insufficiency and in young patients, in whom repeat imaging may be necessary.

MR has superior contrast resolution, which helps detect graft infections in cases that are equivocal with CTA [3]. A time-resolved MR angiography may help demonstrate flow between the aorta and the fistulized organ(s) [11].

However, MR has numerous limitations, including longer acquisition time, the requirement of patient cooperation, and limited availability in emergency scenarios. An adjunctive CT is still required for evaluation of areas with vascular or graft-related calcifications, metallic fixation sites, or the outer metallic surface of the graft. Although the extent of susceptibility artifacts largely depends on the metallic composition of the endografts, all endografts usually produce some level of radiofrequency shielding or “caging” on MR, which may hinder visualization of the graft lumen, irrespective of the material used [3]. Another disadvantage of MR is the potential inability to differentiate perigraft gas from aortic wall calcification.

#### Digital subtraction angiography (DSA)

DSA is rarely the initial imaging modality for interrogation of AoFs. It offers both diagnostic and therapeutic benefits (stent placement and embolization) [4]. DSA has better spatial resolution than CTA, and can evaluate arterial anatomy in greater detail, aiding in surgical planning [3]. It can detect bleeding rates as slow as 0.5 mL/min, and additionally, can aid in the dynamic assessment of vascular pressure gradients [12]. DSA is especially valuable in evaluating patients with prior graft repairs; however, its accuracy is limited when detecting primary AoFs [13].

#### Nuclear medicine imaging

The presence of aortic graft infections is an important cause of septicemia and acts as a catalyst for the development of AoFs [3]. CTA may be unable to differentiate graft infections from postoperative changes, and in such cases, scintigraphy with technetium ^99^m (^99^mTc) hexamethylpropyleneamine oxime–labelled white blood cells (WBCs) or indium ^111^ (^111^ In) oxime–labelled WBCs can be performed [3]. Radiolabelled WBC studies have a specificity ranging from 60 to 100% for the diagnosis of graft infection. For assessing abdominal AoFs, ¹¹¹In-labeled WBCs scintigraphy is preferred over ⁹⁹mTc tracers, as the latter exhibits physiological GI and urinary excretion that can interfere with interpretation [3].

Fluorine 18– fluorodeoxyglucose (FDG) PET-CT offers higher spatial resolution, and is being increasingly used in view of its higher sensitivity and specificity (95% and 80%, respectively) in detecting vascular graft infections [14]. FDG-PET/CT should be performed at least 3–4 months post-surgery/intervention, and prior to the initiation of antibiotic therapy, since post-operative inflammation may yield false-positive results, while antibiotic usage can result in false-negative findings [15].

^99^mTc-tagged red blood cell scan is the most sensitive imaging modality for the detection of slow bleeding as low as 0.05–0.10 mL/min [16]. It is not performed for massive bleeding due to its longer acquisition time. Therefore, it is typically used for detecting bleeds in cases involving a tenuous fistula tract [3], which may be occult on the other imaging modalities.

#### Ultrasonography (US)

US plays a very limited role in the assessment of AoFs. It can be performed in patients with contraindication to the use of iodinated contrast. However, visualization of the deep-seated aorta is often challenging because of overlying bowel gas and unfavourable body habitus [17]. Despite these limitations, point-of-care US has, in isolated reports, demonstrated findings suggestive of aortoenteric fistula, such as heteroechoic mass-like collections contiguous with both the aorta and the bowel, outpouchings from the aorta into the collection, and air foci in the aortic wall or lumen or in the periaortic collection [18]. Findings of an aortocaval fistula on US include turbulent arterialized venous flow [19]. Additionally, contrast-enhanced US can delineate a fistulous tract when contrast is seen passing from the aorta into the adjoining organ [20]. Transesophageal echocardiography may also assist in detecting flow abnormalities involving the aortic arch, root, and proximal descending aorta, and can be performed both intraoperatively and in unstable patients [21].

### Imaging features

The direct and indirect imaging features common to AoFs have been tabulated previously in Table 1. The detailed description and the specific imaging features of each type of AoFs are described in the following sections.

#### 1) Aortoatrial fistula (AAF)

AAF represents an aberrant communication of the aorta with either atrium. AAF can be either acquired (more commonly involves right atrium) [22] or are congenital in origin (congenital aortoatrial tunnels and coronary-cameral fistulas), with the former being more common. Infective endocarditis is the most common cause, often developing in the setting of a prosthetic valve. Aortic aneurysms with or without dissection and complications following iatrogenic procedures such as aortic root or valve surgery are also common causes of AAF (Table 3).

Due to a high-pressure gradient, there is shunting of blood from the aorta into the right or left atrium [22], leading to volume overload and eventually cardiac failure. The symptoms include dyspnea, palpitations, chest pain, cough, fatigue, and cardiogenic shock. In patients who develop a new, continuous murmur or present with sudden-onset cardiac failure, with a history of aortic disease or prior cardiac surgery/intervention, a high index of suspicion for AAF is warranted [23]. Treatment of AAF has to be tailored according to its etiology.

**Table 3:** Acquired causes of aortoatrial fistula

1) Infectious endocarditis - More common with prosthetic valves, with a prevalence of 5.8% (while with native valves, the prevalence is 1.6%) - Most common implicated organism: Staphylococcus
2) Aortic aneurysm
3) Aortic dissection
4) Complications following cardiac surgeries - Aortic root or valve surgery - Coronary artery bypass graft placement - Percutaneous closure of atrial septal defect - Pulmonary vein ablation - Angiography - Insertion of atrial appendage closure device
5) Ruptured sinus of Valsalva aneurysm
6) Trauma

### Imaging features

**Fig. 2:**
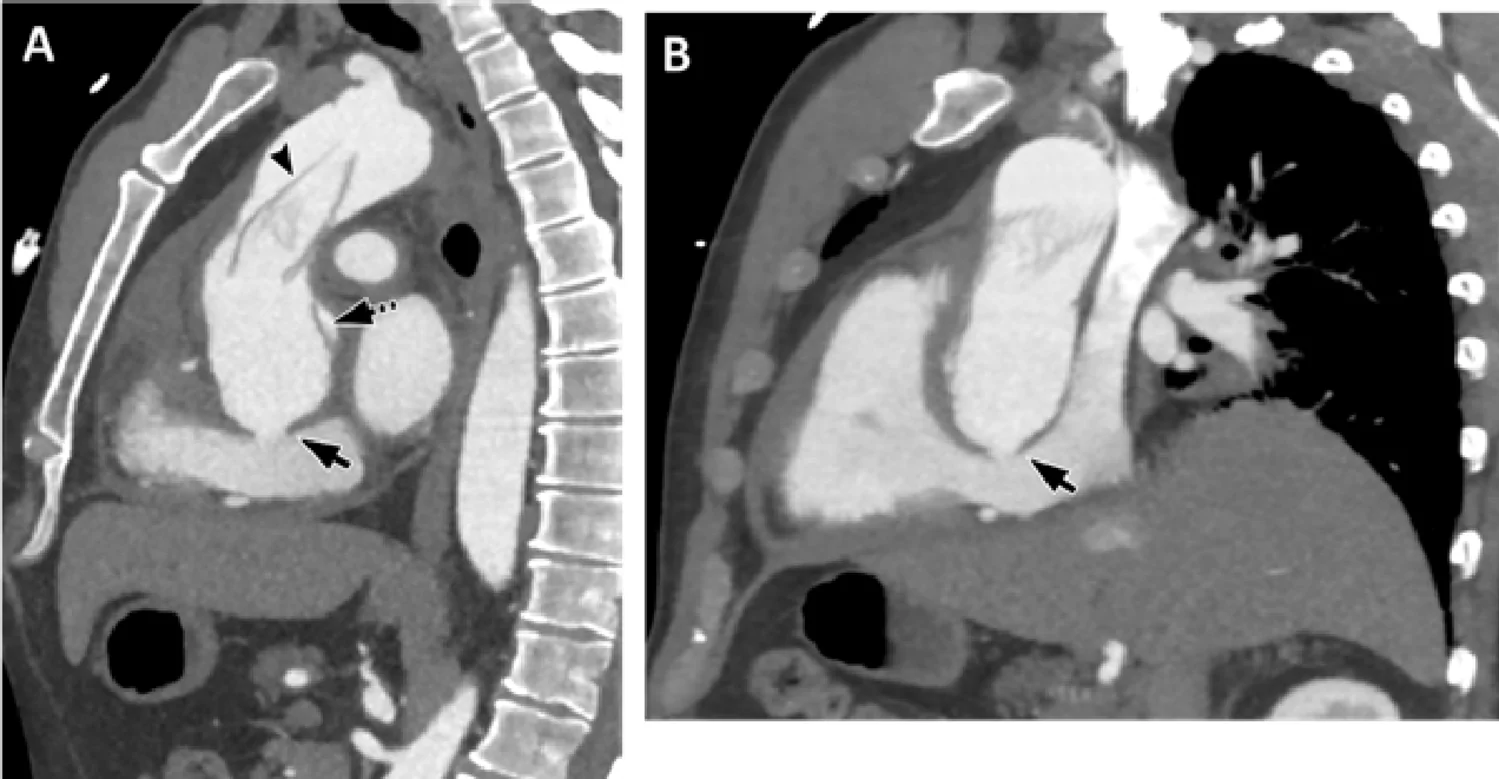
Aortoatrial fistula. A 57-year-old male presented with sudden onset chest pain radiating to his back. Sagittal (**A**) and coronal CT (**B**) images depict type A aortic dissection (arrowhead) beginning in the sinotubular junction (dashed arrow) and extending to the ostium of the right brachiocephalic artery (not shown). Also seen is a fistulous communication between the right atrium and the ascending thoracic aorta (solid arrow). The communication was closed with suturing the felt placed at the edges of the fistula

**Fig. 3:**
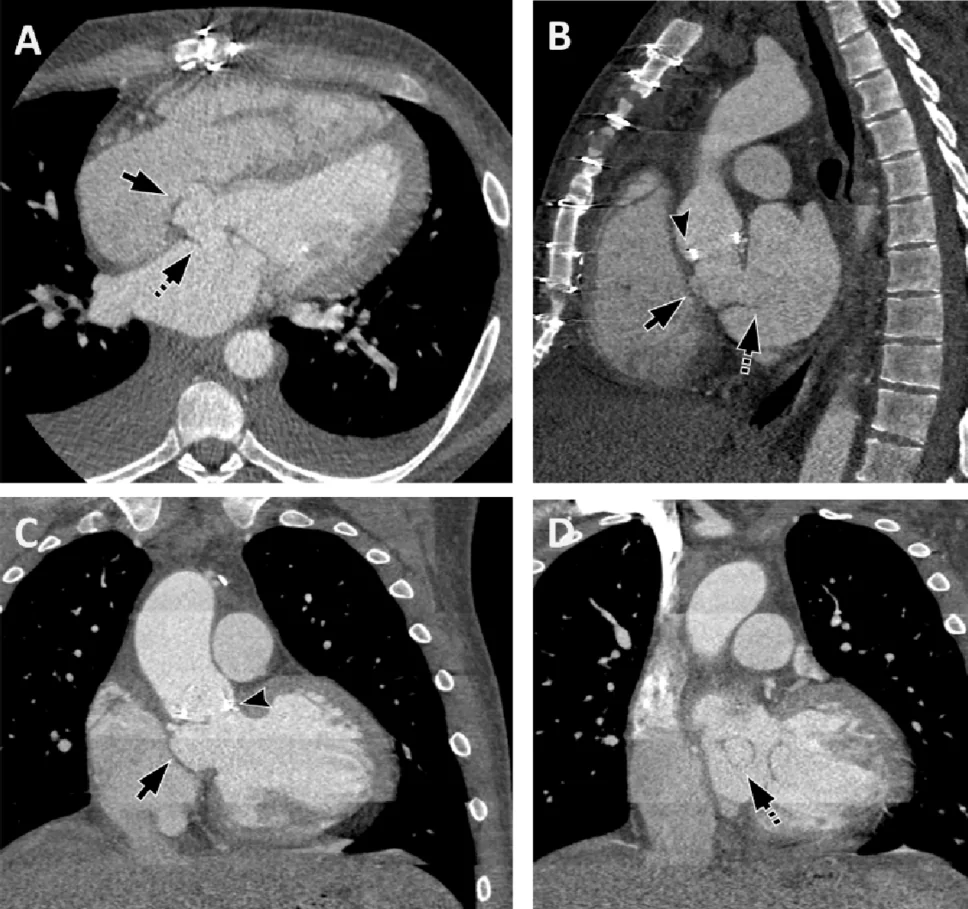
Aortoatrial fistula. A 37-year-old male with a history of valvular endocarditis and post-aortic valve repair. Axial (**A**, **C**, **D**) and sagittal (**B**) CT images depict a focal outpouching arising from the left ventricular outflow tract (solid black arrows), underneath the prosthetic aortic valve (arrowhead), compatible with a contained rupture. This courses slightly posterolateral into the left atrium, resulting in a fistula between the left ventricular outflow tract and left atrium (dashed arrows)

Various imaging modalities provide complementary information for the diagnosis and management of suspected AAF. Both CTA and cardiac MRI provide an excellent overview of the extracardiac anatomy, serving as a roadmap for surgical planning. On CTA (Figs. 2 and 3), direct imaging features of AAF include contrast extravasation from the aorta into the atrial chamber. In cases of aortic dissection leading to AAF, a tract connecting the true or false aortic lumen to the atrium may be visualized. Indirect imaging features are more common and include adhesions and fibrosis between the aorta and the atrium, features of endocarditis, and aortic root dilatation.

Cardiac MRI is the preferred investigation to quantify the cardiac shunt fraction and flow measurements [24]. Advanced techniques, such as four-dimensional flow MRI, can be utilized for better characterization of AAF [25]. However, limitations of MRI include its longer scan time (precluding its use in unstable patients), inability to be used intra-operatively (in contrast to echocardiography), and requirement of gadolinium-based contrast administration.

Three-dimensional trans-esophageal echocardiography (TEE) provides excellent spatial resolution and visualization of the fistula and its relation with surrounding organs [26] and it can be performed both at bedside in unstable patients as well as for intraoperative assessment [21].

Cardiac catheterization can help with shunt quantification. However, catheterization is invasive, and flow measurements tend to be less accurate compared to cardiac MRI [24].

#### 2) Aortopulmonary fistula (APF)

APF is an extremely rare condition [27]. APF can occur as a part of congenital heart disease. On the other hand, causes of acquired APFs include rupture of a thoracic aortic aneurysm into the pulmonary artery—commonly secondary to aortic dissection—along with prior aortic or pulmonic valve interventions, trauma, and sepsis.

APF leads to the development of an acute left-to-right shunt (with a continuous murmur), and patients usually present with pulmonary edema and acute cardiac decompensation [28]. APF should be suspected if a patient with a thoracic aortic aneurysm presents with acute cardiac failure [29].

Treatment options for APF include surgical repair, endovascular coil embolization, and [30] stent-graft placement [31]. Surgery is the preferred option currently; however, the use of endovascular methods is increasing due to their less invasive nature and shorter recovery periods [32].

### Imaging features

**Fig. 4:**
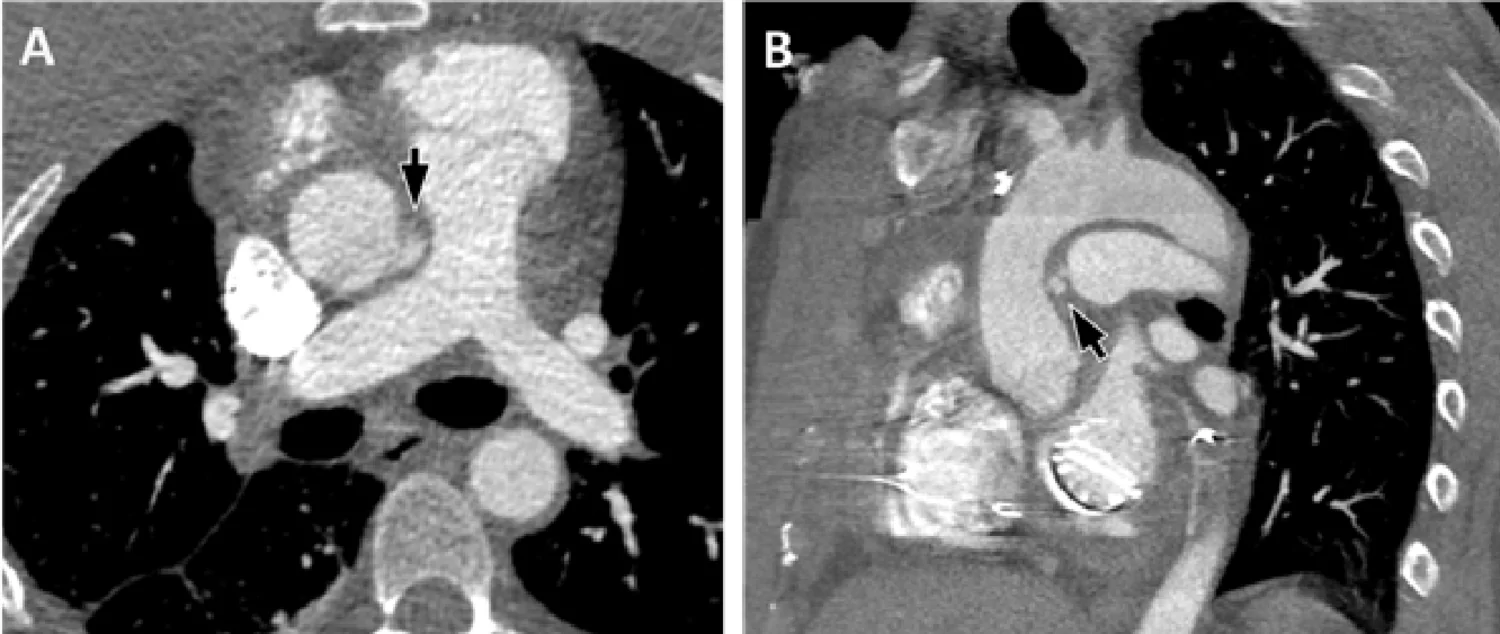
Aortopulmonary fistula. Postcontrast axial (**A**) and coronal oblique (**B**) CT chest images in a 41-year-old male, post mitral valve replacement, demonstrate an abnormal fistulous communication between the ascending thoracic aorta (arising from its left lateral aspect) and the adjacent main pulmonary artery, compatible with an aortopulmonary fistula (black arrow)

Transthoracic echocardiography (TTE) is often the initial investigation for APF. TTE may show a continuous, turbulent, high-velocity flow between the right ventricular outflow tract/pulmonary artery and the aorta [33]. High oxygen saturation in the pulmonary arterial blood and an oxygen step-up between the right atrium and the pulmonary artery, as determined during cardiac catheterization, are confirmatory [29]. CTA (Fig. 4) may show contrast extravasation from the thoracic aorta into the pulmonary artery or nonspecificfeatures of aortic dissection and thoracic aortic aneurysm. Cardiac MRI can help in the quantification of flow through the shunt/APF.

#### 3) Aortobronchial fistula (ABF)

ABF is a rare entity. Its incidence is underestimated, with 30% cases diagnosed at autopsy [34]. It typically occurs between the left bronchial tree (usually the membranous part) and the descending thoracic aorta, due to their anatomical proximity [35]. The various causes of ABF are tabulated in Table 4.

Hemoptysis of varying severity is the most typical symptom, but it is not specific [36]. Other symptoms may include chest pain and dyspnea. Patient may present clinically years after the thoracic procedures (mentioned in Table 4) [37]. Therefore, any patient who presents with recurrent and massive hemoptysis with a history of thoracic aorta disease or any prior thoracic surgeries warrants consideration of ABF. ABF has a high mortality rate, especially if there is a recurrence of hemoptysis secondary to clot dislodgement [35].

Endovascular treatment with deployment of an aortic stent graft is preferred over open surgery. However, a few of its limitations include graft contamination, leakage, and graft migration [5].

**Table 4:** Causes of aortobronchial fistula [3, 38]

1) Prior surgeries: ¬ Esophagectomy ¬ Thoracic aorta surgeries ¬ Upper aerodigestive tract surgeries ¬ Childhood cardiac surgeries (e.g., tetralogy of Fallot, patent ductus arteriosus repair)
2) Prior interventions: ¬ Endobronchial stent placement ¬ Endobronchial blocker placement (for preferential airway aeration)
3) Thoracic aortic disease: ¬ Dissection ¬ Aneurysm ¬ Pseudoaneurysm ¬ Traumatic injury
4) Radiation therapy (for neck or lung malignancies)
5) Vasculitis (Takayasu arteritis)

### Imaging features

**Fig. 5:**
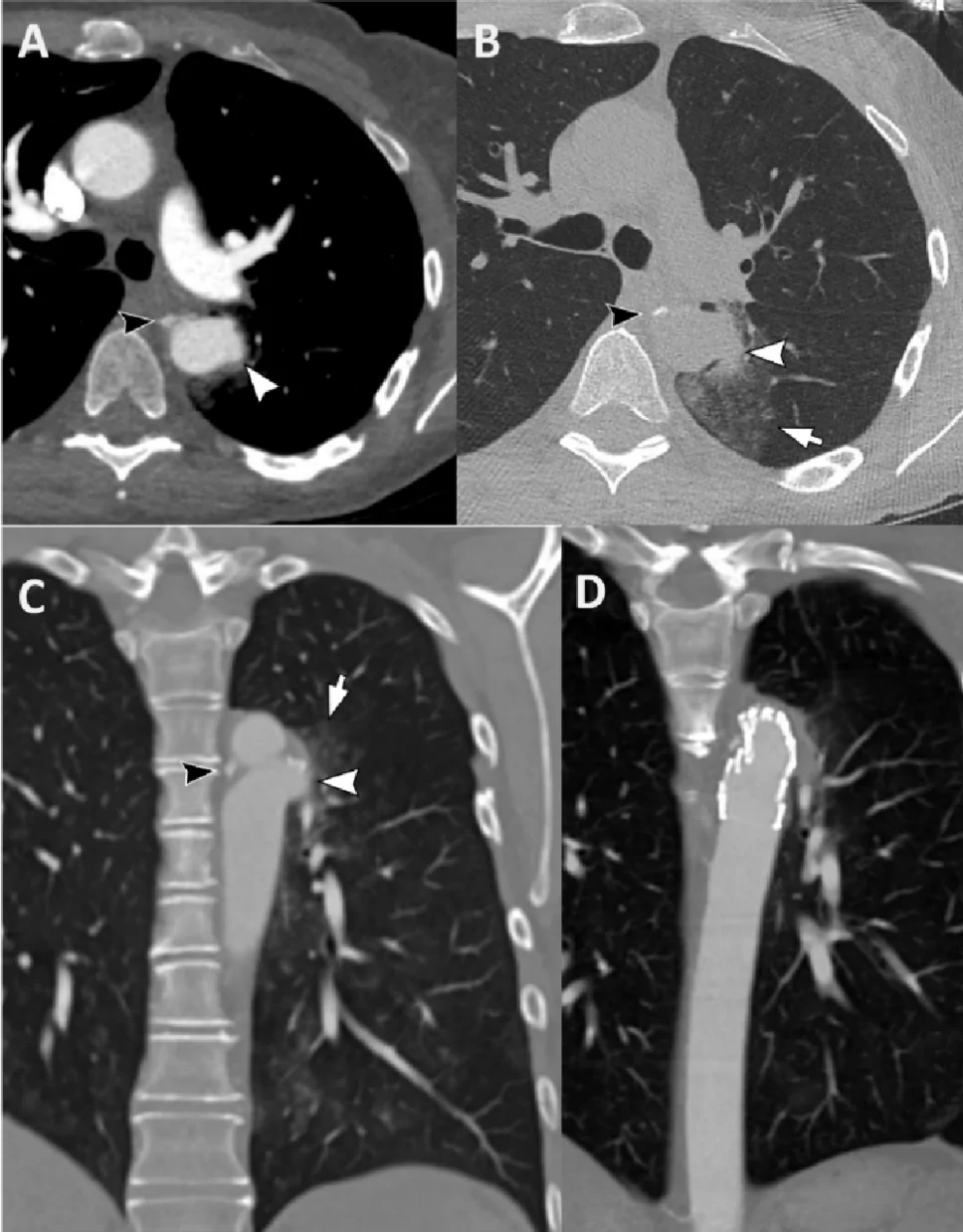
Aortobronchial fistula. A 53-year-old female with a history of repair of aortic dissection secondary to motor vehicular collision 20 years ago, presented with massive hemoptysis. Axial CTA chest (**A**) shows a pseudoaneurysm (white arrowhead) at the level of the distal anastomosis of the prior interposition graft, with a concern for impending rupture. The black arrowhead in (**A**, **B**, and **C)** depicts prior suture material. Axial (**B**) and coronal (**C**) CT chest in lung window show the anastomotic site pseudoaneurysm (white arrowhead) surrounded by ground-glass opacities (white arrow) suggestive of pulmonary hemorrhage. The hemoptysis was likely secondary to a fistula between the aortic pseudoaneurysm and the airways (aortobronchial fistula). Posttreatment coronal CT (**D**) performed 2 days later shows aortic stent, complete exclusion of the pseudoaneurysm, and near resolution of adjacent ground-glass opacities

**Fig. 6:**
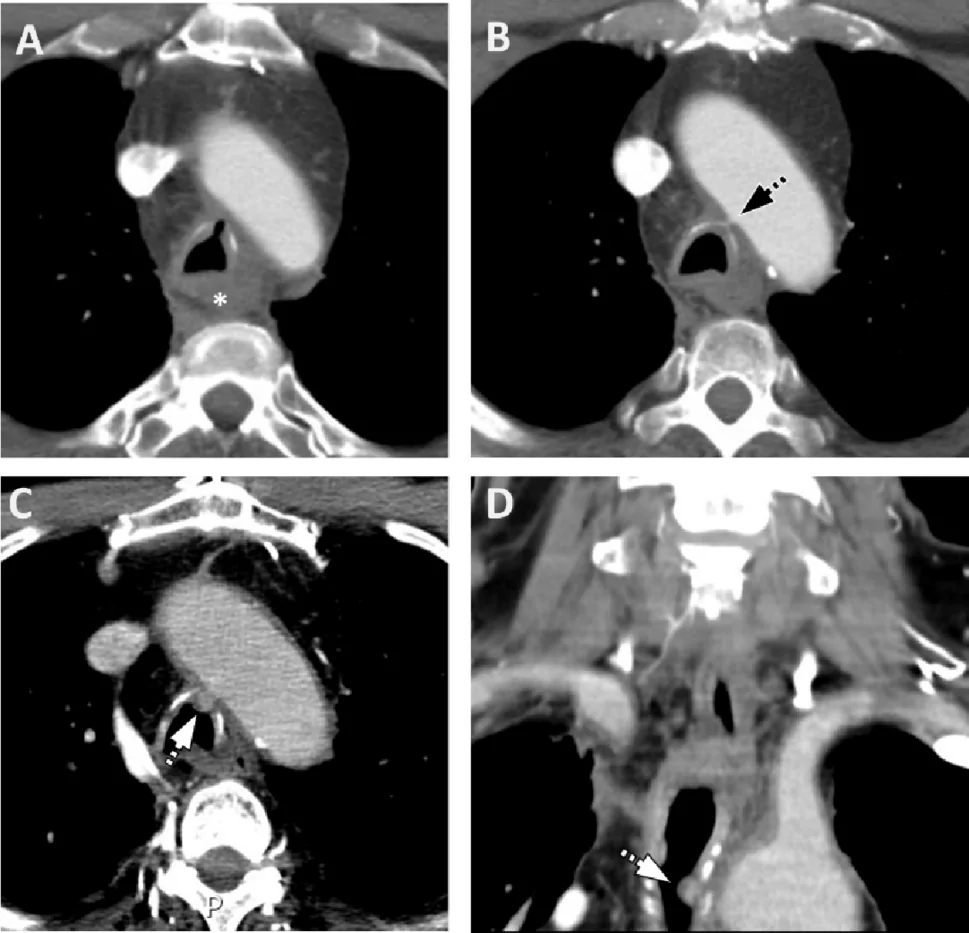
Aortotracheal fistula. (**A)** Postcontrast CT chest in a 51-year-old male shows treatment changes of radiotherapy (asterisk) around the trachea. Two months later, the patient presented with massive hemoptysis. (**B)** Axial postcontrast CT shows the focal outpouching of the aortic arch (dashed black arrow). (**C)** Postcontrast axial and **D** coronal CT show a pseudoaneurysm arising from the aortic arch within the tracheal lumen (aortotracheal fistula) (dashed white arrow), which resulted in massive hemoptysis

ABF can be very difficult to diagnose, and repeated imaging might yield negative results in view of the intermittent nature of bleeding, similar to other AoFs. Chest radiograph may show focal aortic enlargement with or without adjoining air space opacification (secondary to pulmonary hemorrhage). CTA (Figs. 5 and 6) may show indirect signs such as aortic outpouching or penetrating ulcer or dissection and ill-defined tissue planes between the aorta and the bronchus. The adjacent lung shows ill-defined, focal ground-glass opacities suggestive of pulmonary hemorrhage. When the blood is aspirated, there can be ground-glass opacities or tree-in-bud nodules in the dependent segments of the lung. Direct signs including active contrast extravasation into the bronchial tree are extremely uncommon. Tsuchiya et al. described the presence of a dilated, air-filled bronchiole adjacent to perigraft air within the region of atelectasis in all 6 of their patients with confirmed ABF [39]. Bronchoscopy may help in localizing the source of bleeding, but fails to identify the cause of ABF [40].

#### 4) Aortoesophageal fistula (AEoF)

The most common site of development of an AEoF is between the descending thoracic aorta and the middle-third of the esophagus due to their close proximity [1]. In contrast to other AoFs, AEoF are more commonly primary in origin. In the decreasing order of frequency, the common causes for the development of AEoF include rupture or repair of thoracic aortic aneurysm (54%), foreign body ingestion (19%), and esophageal malignancies (17%) [41]. Other causes include prior thoracic aorta/esophageal surgery or graft placement, prior radiotherapy, infections (syphilis, tuberculosis), and ablation in atrial fibrillation.

AEoF is classically associated with the Chiari triad, that comprises mid-thoracic pain (or dysphagia), a small sentinel bleed, and massive fatal hematemesis following an asymptomatic interval [42]. The goal of imaging is to identify AEoF before the final exsanguinating event. The time interval between the herald bleed and fatal hemorrhage varies and can range from hours to days. This lag is secondary to a combination of hypotension, arterial spasm, and partial occlusion of AEoF by periaortic hematoma [43]. Unlike more common causes of upper GI bleed (such as esophageal or gastric varices), AEoF usually presents with bright red blood [44]. Hence, in patients with hematemesis comprising bright red blood and a history of aortic aneurysms or aortic/esophageal surgery, a high index of suspicion for AEoF should be maintained. Untreated AEoF has a very high mortality rate of 76% [45].

For treatment of AEoF in the acute setting, the Sengstaken-Blakemore tube can temporarily achieve hemostasis by its tamponade effect [46]. However, its complications include aspiration, esophageal perforation, and broncho-esophageal fistulas [47]. Definitive treatment options include thoracic endovascular aortic repair (TEVAR) and open surgery.

### Imaging features

**Fig. 7:**
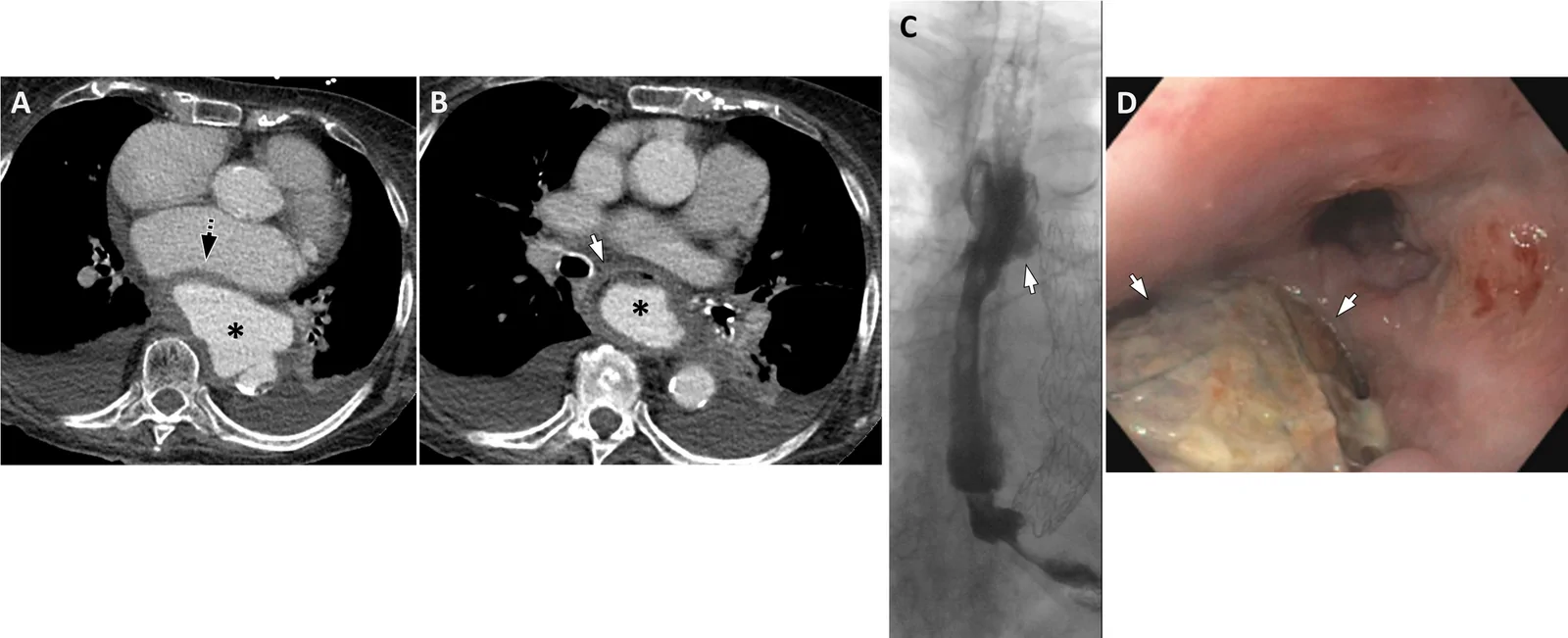
Aortoesophageal fistula. An 89-year-old female presented with dysphagia and upper gastrointestinal (GI) bleeding. Axial contrast-enhanced CT images (A, B) shows a large saccular aneurysm of the descending thoracic aorta (asterisk), with mass effect on the left atrium (dotted black arrow, A) and esophagus (white arrow, B) , and loss of intervening fat planes. Also noted are bilateral small-volume pleural effusions. Fluoroscopic image (C) after aortic stenting both the descending thoracic aorta and the esophagus shows leakage of oral contrast at the site of fistula (white arrow). Upper GI endoscopy 6 weeks later, after removal of the esophageal stent, demonstrates a persistent fistula, and visualization of the aortic stent (arrows)

CT may show indirect signs such as aortic or esophageal outpouching, mediastinal collections, extraluminal air, esophageal wall thickening, and aortic wall discontinuity with loss of adjoining fat planes (Fig. 7). Direct signs of AEoF on CTA (i.e., extravasation of intravenous contrast material into the GI tract or leakage of orally administered contrast into the periaortic space) are rarely encountered. Non-contrast CT is invaluable for identifying ingested hyperdense foreign bodies and evaluating confounding calcifications [48].

Upper GI endoscopy (UGIE) is commonly performed to evaluate upper GI bleeding and may show a bulging pulsatile lesion, submucosal hematoma, or an exposed aortic stent [49]. UGIE can be hazardous, as it may lead to dislodgement of a partially occlusive periaortic hematoma, precipitating fatal hemorrhage [45].

#### 5) Aortoenteric fistula (AEF)

**Fig. 8:**
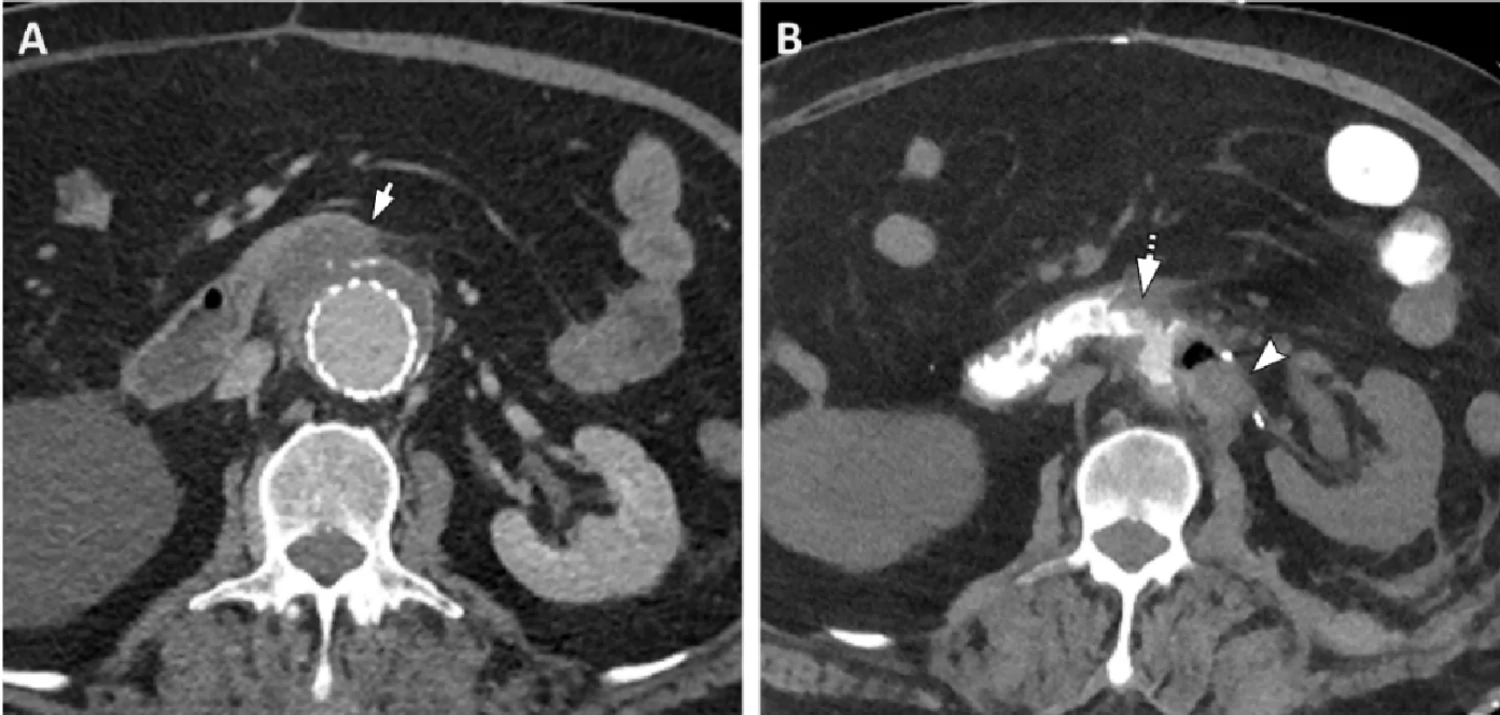
Secondary Aortoduodenal fistula. A 76-year-old male with a history of abdominal aortic graft placement 5 years ago for abdominal aortic aneurysm presented with abdominal pain, preceded by right knee joint septic arthritis. Axial CT (**A)** shows a small collection with subtle adjacent stranding (white arrow) between the third part of the duodenum and the aorta. A few days later, there was hematemesis and melena, and an aortoduodenal fistula was suspected. Hence, the graft was removed and replaced with a homograft. Twelve hours later, the patient became hypotensive with increasing abdominal pain. Noncontrast CT with positive oral contrast (**B)** demonstrates leakage of contrast (dashed white arrow) from the third part of the duodenum around the aortic homograft (arrowhead)

**Fig. 9:**
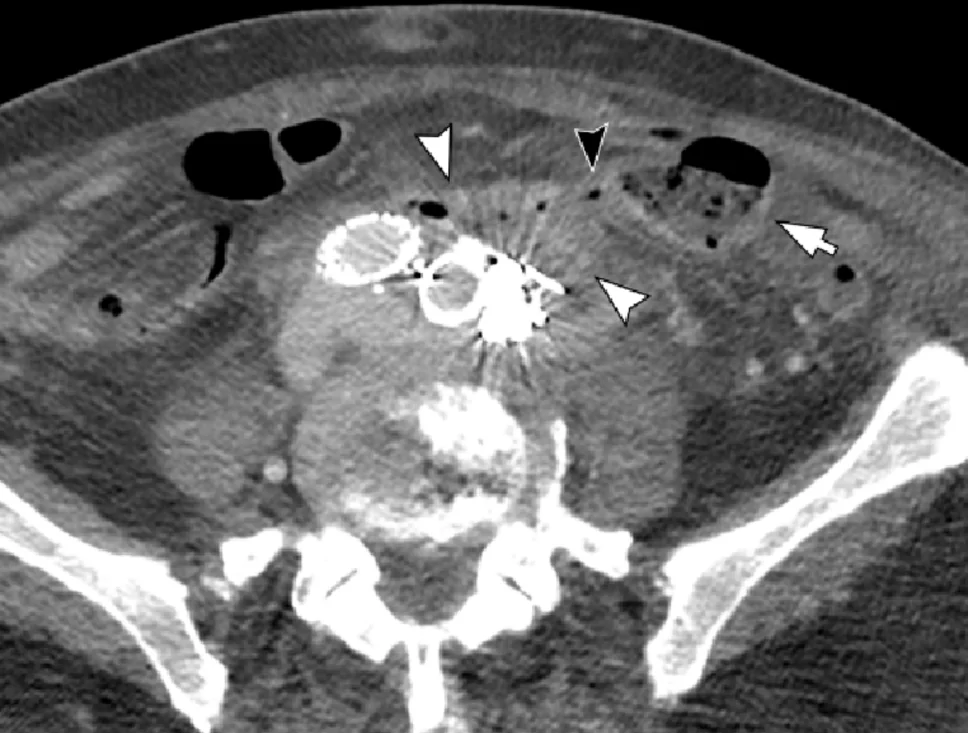
Aortocolic fistula. An 84-year-old male with a history of EVAR for a contained rupture of abdominal aortic aneurysm (AAA) 6 weeks ago, was incidentally found to have air foci around the graft on a CT, concerning for an infection. Two days later, he developed bright red blood per rectum (BRBPR), prompting another CT examination. CECT abdomen shows an air-containing tract (black arrowhead) arising from a loop of colon (white arrow) leading to an air-containing collection surrounding the graft (white arrowheads). An emergent EVAR repair, AAA repair with segmental sigmoidectomy was performed

Primary AEF is rare and is usually seen with an atherosclerotic abdominal aortic aneurysm [50]. Secondary AEF is the overall most common subtype of AoFs, with a prevalence of 0.5–2.3% [3]. Secondary AEF typically occurs as a complication of aortic aneurysm repair. Up to 80% of secondary AEF involve the third and fourth portions of the duodenum (Fig. 8), followed by jejunum, ileum, and colon (Fig. 9) [51, 52]. The close anatomical proximity between the juxtarenal and infrarenal aorta and the retroperitoneally fixed duodenum renders this region particularly susceptible. Chronic low-grade graft infection (gut flora colonization), foreign body reaction due to grafts/sutures, and repetitive pressure on the intestine from aortic pulsations are common predisposing factors.

Other less common causes of AEF include tuberculosis, syphilis, mycotic infection, collagen vascular diseases, radiation, ingested foreign bodies, pancreatitis, and ballistic injuries.

Clinical features, in descending order of occurrence, include GI bleeding, sepsis, abdominal pain, back pain, groin pain, and pulsatile abdominal mass [2]. GI bleeding can present as an initial small sentinel or herald bleeding followed by exsanguination. Hematochezia and bright red blood per rectum are uncommonly seen. Cooper’s triad, seen in up to a quarter of primary AEF patients, includes upper GI bleed, abdominal pain, and a pulsatile abdominal mass [53]. Patients without bleeding can be difficult to diagnose, and AEF should be considered in patients with a history of aortic surgery who present with fever of unknown origin (even without evidence of GI bleeding).

Treatment options for AEF include endovascular aortic repair (EVAR) and open surgery. Surgery is the preferred option, has a lower rate of sepsis (due to thorough debridement of infected tissues), but has a higher hospital mortality rate. EVAR can be performed upfront in primary AEF without any signs of infection; otherwise, it can be used as a bridging therapy [54].

### Imaging features

CT can demonstrate both direct and indirect imaging features similar to other AoFs. Since advanced perigraft infection can produce AEF, it may be challenging to differentiate between the two entities. CT findings that favor AEF over graft infection include ectopic gas, focal bowel wall thickening, breach of the aortic wall, and extravasation of contrast material into the bowel lumen [2]. Furthermore, if GI bleeding is the predominant clinical presentation, the presence of any CT findings suggestive of a perigraft infection should alert the radiologist to a potential secondary AEF. Finally, the persistence of perigraft air beyond a month after surgery or the persistence of perigraft soft-tissue thickening, fluid, or hematoma more than 2–3 months after the procedure should raise a suspicion of secondary AEF.

Nuclear medicine scintigraphy with ^99^mTc labelled red blood cells can be used to differentiate graft infection from AEF. The presence of radiotracer within the bowel lumen is highly suggestive of AEF.

Endoscopy is of limited value in evaluating AEF, as most AEFs occur in the third and fourth parts of the duodenum, and upper GI endoscopy usually only reaches the second segment of the duodenum [55]. Further, it is difficult to perform endoscopy in hemodynamically unstable patients, which is a commonly encountered presentation of AEF.

#### 6) Aortocaval fistula (ACF)

ACF occurs in approximately 2%–7% of cases, although prevalences of up to 10% have also been reported [3]. The most common cause (80%) of ACF is AAA, and it occurs in 1% of AAA patients and 4% of patients with ruptured AAA [56]. Other relatively common causes of ACF include penetrating or spinal trauma (with thoracolumbar translation) and spinal surgery or exploratory laparotomy. Rare causes include inferior vena cava (IVC) filter placement, IVC dissection, mycotic aneurysm, Takayasu arteritis, and connective tissue disorders [57]. The infrarenal aorta is the most common site for the formation of ACF [3].

Clinical presentation of ACF depends on the fistula size [58] and its chronicity [59]. Owing to left-to-right shunting, patients may present with high-output cardiac failure, lower limb venous congestion/swelling, deep venous thrombosis, arterial insufficiency, and pulmonary embolism [59]. Shock is rarely seen, as blood remains intravascular [3]. Common non-specific symptoms include acute abdominal and back pain, dyspnea, and a pulsatile abdominal mass with a bruit.

Treatment options include open surgery and EVAR.

## Imaging features

**Fig. 10:**
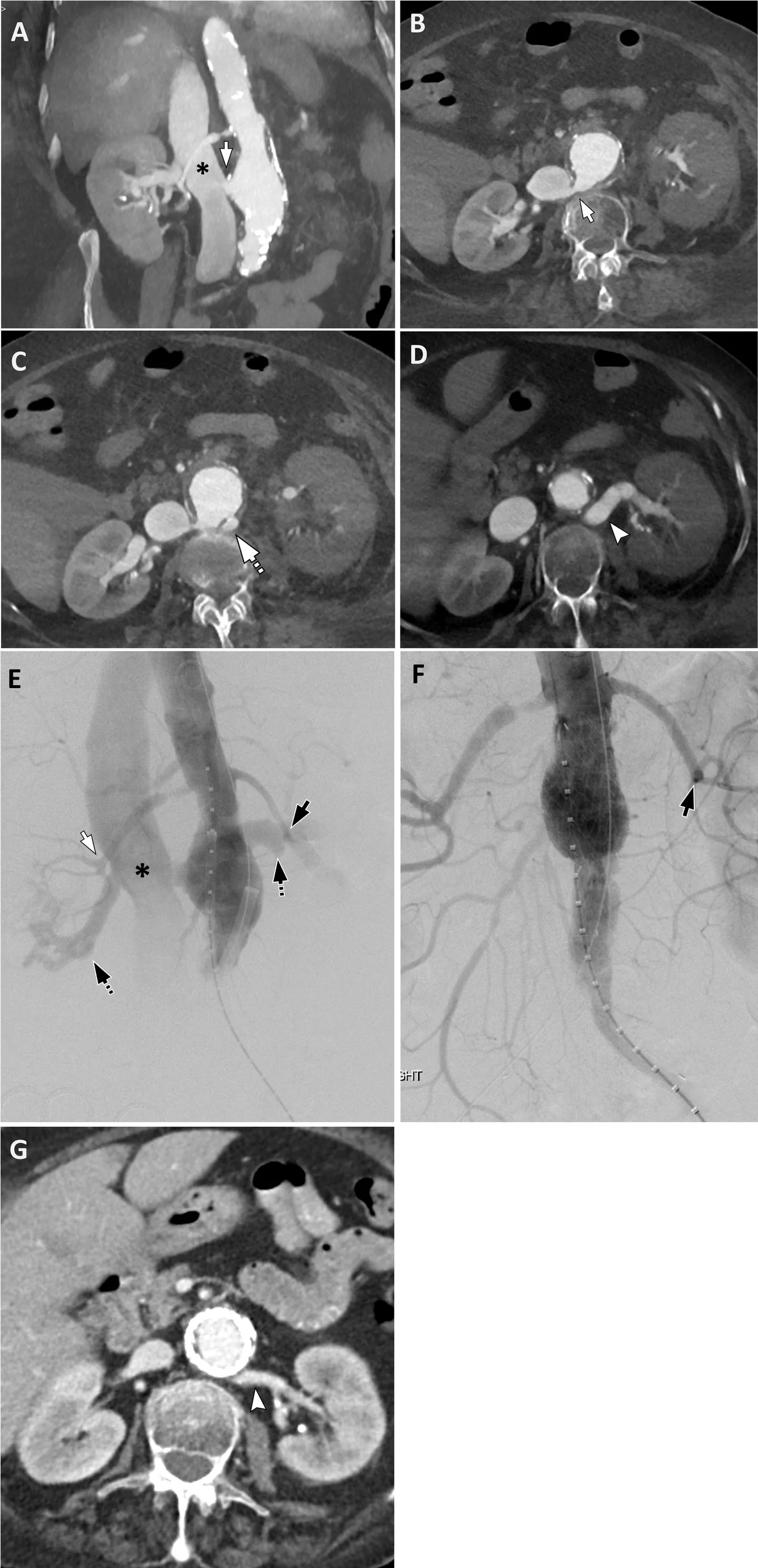
Digital subtraction angiogram (DSA) image (**E**) shows apparent pruning off of the left renal artery (black arrow), with early filling of bilateral renal veins (dashed black arrows) and IVC (asterisk). Compare with the normal branching of the right renal artery (white arrow).Aortocaval fistula. A 66-year-old female presented with an acute onset of left flank pain. She developed acute kidney injury, with a creatinine of 3.5 mg/dL. Ultrasound did not show any hydronephrosis. Four days later, she developed feeble pulses, pain and discoloration of the toes. Coronal maximum intensity projection (MIP) arterial phase image (A) and axial MIP arterial phase image (B) show aortocaval fistula (white arrow) with early opacification of the inferior vena cava (IVC, asterisk). There is an additional fistula between the aorta and left renal vein (dashed white arrow, C), with a dilated and tortuous left renal vein (arrowhead, D), causing increased intrarenal pressure and hence poor contrast opacification of the left kidney. Digital subtraction angiogram (DSA) image (E) shows apparent pruning off of the left renal artery (black arrow), with early filling of bilateral renal veins (dashed black arrows) and IVC (asterisk). Compare with the normal branching of the right renal artery (white arrow). DSA image after aortic stenting (F) shows the normal opacification of the left renal artery and its branches (black arrow) and a normal nephrogram without early opacification of renal veins and IVC. Contrast CT axial image after aortic stenting (G) shows a normal-caliber left renal vein (arrowhead) and IVC with symmetric enhancement of both kidneys

**Fig. 11:**
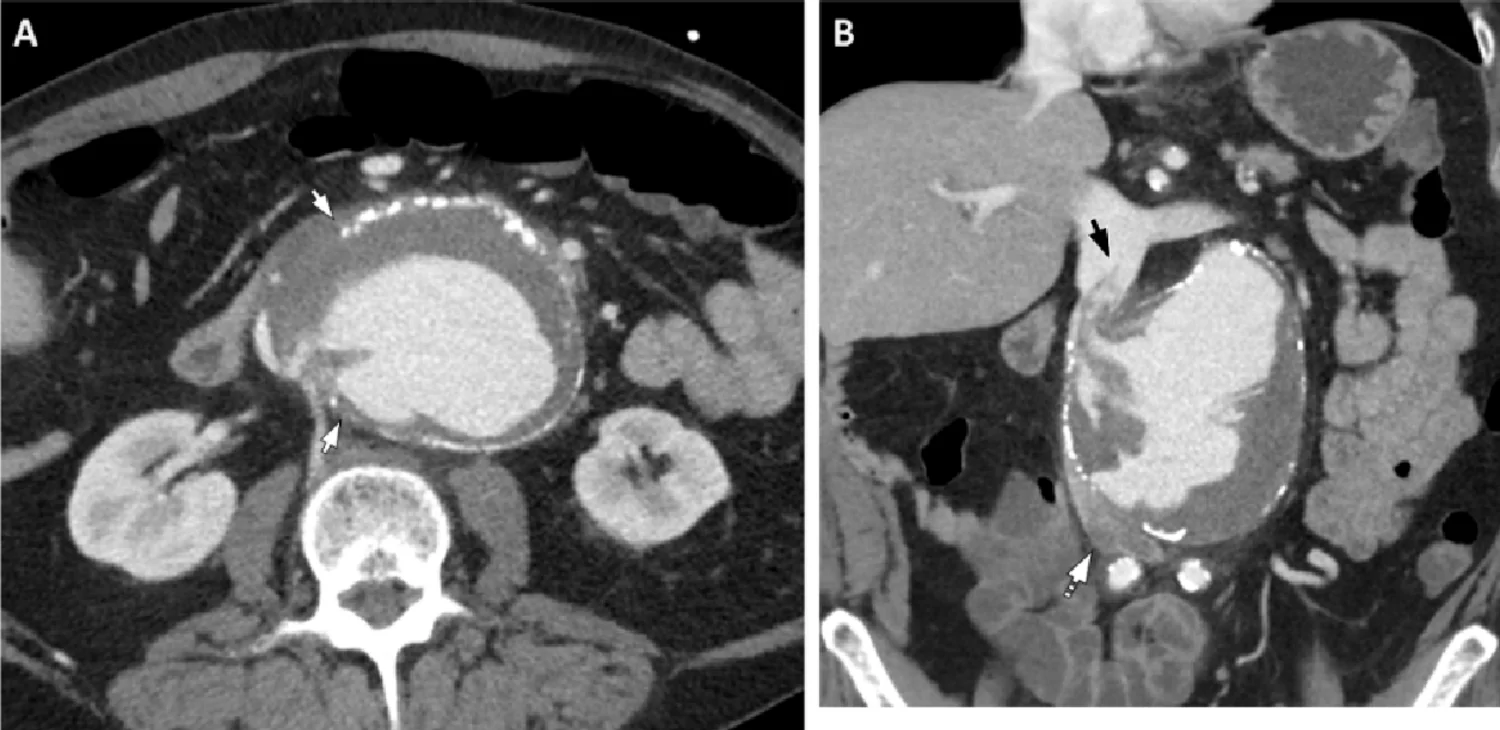
Aortocaval fistula. A 70-year-old male presented with sudden onset abdominal pain, hypotension, and a pulsatile abdominal mass. Axial contrast-enhanced CT (**A**) shows a large aortic aneurysm with peripheral, partially calcified, atheromatous plaque. There is a loss of rounded contour and an interruption of calcific plaque (white arrows) between 7 and 10 o’clock, with loss of interface with the IVC. Coronal reformatted image (**B**) shows a fistula between the infrarenal IVC and aorta, with floating aortic atheromatous plaque contents into the IVC (black arrow). IVC inferior to the fistula (dashed white arrow) is not well opacified

Simultaneous opacification of the aorta and the IVC in the arterial phase is pathognomonic (Figs. 10 and 11). However, in cases with AAA, the IVC may be compressed, and the enhancement of the IVC may be difficult to evaluate [60]. In such cases, opacification of renal or iliac veins in the arterial phase and loss of the aortocaval fat plane can be suggestive of ACF [59, 60]. The reflux of contrast from the right atrium should not be mistaken for ACF, as it is of less attenuation [60]. Additional imaging features of ACF include the presence of retroperitoneal hematoma, delayed enhancement of the renal cortex [60], and delayed opacification of the femoral arteries [58]. Acquisition of the angiographic phase during expiration may theoretically improve visualization of suspected aortocaval fistulae by increasing inferior vena cava calibre, given the known respiratory variation in caval diameter.

## Conclusion

Aortic fistulas are life-threatening entities associated with high morbidity and mortality. With the rising use of endovascular interventions, aortic fistulas should be kept in the list of differential diagnoses in patients who present with gastrointestinal bleeding or hemoptysis with a prior history of aortic interventions. Since clinical signs may not be sufficiently specific, imaging is usually required to establish an accurate diagnosis, with CT angiography being the preferred imaging modality. Direct visualization of the fistulous tract on imaging is, however, rare. Hence, familiarity with the more common secondary (indirect) imaging features enables the radiologist to identify the aortic fistulas, thereby improving patient care.

## Data Availability

No datasets were generated or analysed during the current study.
